# Indigenous people in Aotearoa New Zealand are overrepresented in cannabis convictions

**DOI:** 10.1186/s12954-022-00613-9

**Published:** 2022-03-17

**Authors:** Wetini Rapana, Taylor Winter, Ririwai Fox, Benjamin C. Riordan, Rajas Kulkarni, Waikaremoana Waitoki, Damian Scarf

**Affiliations:** 1 95a Union Place East, Goddard Laboratory Building, Department of Psychology, University of Otago, Dunedin, New Zealand; 2 Department of Psychology, Victoria University of Wellington, Wellington, New Zealand; 3 Centre for Alcohol Policy Research, La Trobe University, Melbourne, Australia; 4 Te Pua Wānanga ki te Ao Faculty of Māori and Indigenous Studies, University of Waikato, Waikato, New Zealand

**Keywords:** Racism, Cannabis, Decriminalisation, Drug policy, Cannabis policy, Population data

## Abstract

**Background:**

Previous work has demonstrated that cannabis laws have had a disproportionate impact on Māori, the Indigenous people of Aotearoa New Zealand. In 2019, the New Zealand Government amended cannabis laws, providing police with the power to determine whether a therapeutic or health-centred approach would be more beneficial than a conviction. In the current study, we use population level data to assess whether this law change has ameliorated the bias in cannabis convictions for Māori.

**Methods:**

Data were drawn from the Integrated Data Infrastructure (IDI), a large government database hosted by Aotearoa New Zealand’s national statistics office. In the IDI, we selected individuals who (1) were between 18 and 65, (2) were Māori or Pākehā (New Zealanders of European descent) and, (3) had any cannabis charges that proceeded to the courts.

**Results:**

Māori ethnicity was a significant predictor of the odds of receiving a cannabis conviction for Māori males (Odds: 1.56), with a marginally significant effect for Māori females (Odds: 1.57). Further, for Māori, there was no reduction in the number of cannabis charges before vs. after the amendment to cannabis laws.

**Conclusion:**

The current study demonstrates that the bias in cannabis convictions for Māori remain. Given this, the New Zealand Government must follow other countries around the world and move forward on cannabis law reform.

## Introduction

The academic literature on disparities in drug convictions has, in large part, focused on the United States (US). This is likely due to the fact the US has the largest prison population in the world, one that imprisons African Americans at nearly triple their proportion of the US population (33% vs. 12%) [9, 17, 25]. Research on these race-based disparities should continue; however, there is a pressing need to increase research on the impact of drug laws on Indigenous populations. For example, Māori, the Indigenous people of Aotearoa New Zealand, are imprisoned at more than triple their proportion of the population, and drug offences account for over half of these convictions [32]. The focus of this paper is  on cannabis convictions as (1) there is growing evidence suggesting that cannabis laws have a disproportionate impact on Indigenous populations and, (2) the legal status of cannabis is going through a rapid period of change in many countries. The obligation to involve Indigenous populations in these legislative changes is clearly articulated in the United Nations Declaration on the Rights of Indigenous Peoples. In Aotearoa New Zealand, these rights are also enshrined in Te Tiriti o Waitangi.

To date, Owusu-Bempah and Luscombe’s [19] work on arrest rates in Canada is one of the only academic studies that utilises population level data to demonstrate that cannabis laws have a disproportionate impact on Indigenous peoples. Surprisingly, the majority of work on cannabis arrest rates and Indigenous peoples has come from freedom-of-information requests submitted by news organisations. Specifically, work by the Toronto Star and Vice in Canada [2, 23], the Guardian in Australia [14], and the Herald in Aotearoa New Zealand [4, 5]. In Aotearoa New Zealand, Māori experienced settler-colonisation across political, economic, cultural and social activities. First, we will provide a brief history of Māori and cannabis laws in Aotearoa New Zealand. Second, we use data from the Integrated Data Infrastructure (IDI), a large government database hosted by Aotearoa New Zealand’s national statistics office, to demonstrate that Māori continue to be disproportionally impacted by the current cannabis laws in Aotearoa New Zealand.

### Māori and cannabis laws in Aotearoa New Zealand

Māori migrated to Aotearoa New Zealand around the fourteenth century, after successfully navigating a 3000 km journey across East Polynesia, to the South Pacific [31]. While also maintaining their ancestral voyaging traditions, Māori social, political and horticultural systems became intricately connected to, and shaped by the new lands [22]. After settling into tribal groupings Māori began trading with other tribes, who had also migrated to Aotearoa New Zealand at different times, with the resources that were readily available in their own regions. At this time, and up until the arrival of Europeans, Māori were not known to use intoxicants [6].

With the arrival of Europeans in Aotearoa New Zealand came both tobacco and alcohol [6]. From the outset, settlers took advantage of the Māori trading economy, exchanging alcohol and tobacco for land, and using tobacco to incentivise the signing of Te Tiriti o Waitangi [18]. Te Tiriti o Waitangi (Treaty of Waitangi) was signed between many of the Māori tribal chiefs and the British Crown. The signing of the Treaty was effectively the starting point for British governance. The oppression of Māori continued unabated after the signing [7, 12]. The increasing confiscation of land forced many Māori from their traditional rural homes to urban townships in pursuit of work [29]. This urban drift was associated with many Māori coming into contact with psychoactive substances—including cannabis—for the very first time [28].

Māori first coming into contact with cannabis (~ 1970s) coincided with the introduction of the Misuse of Drugs Act 1975 [27]. The Misuse of Drugs Act meant that the cultivatation, distribution, possession, and use of cannabis was illegal. Before investigating the impact of the Act on Māori, it is important to highlight the fact that, before the imposition of the Crown’s laws, Māori were governed by principles of tikanga, which can be loosely defined as a collective understanding of right-action [15]. For each area, activity, or environment, there existed a protocol which was mutually understood and collectively reinforced. Breaches of tikanga were believed to strongly invoke negative consequences. Without the imposition of Crown law, it is reasonable to suppose that Māori would have developed tikanga for the safe use of cannabis.

As noted above, the Misuse of Drugs Act made possessing any amount of Cannabis illegal. Police, however, were somewhat lenient in their enforcement [1]. From the outset, this leniency was used in a discriminatory fashion, with Māori markedly more likely to be convicted for the possession of cannabis than non-Māori [8, 11]. Despite its disproportionate impact on Māori, cannabis laws in Aotearoa New Zealand are still governed by the Misuse of Drugs Act 1975. Without the specific aim of addressing it’s impact on Māori, the Government made an amendment to the Act in 2019 [16]. In essence, the amendment instructs police to determine whether a health-centred or therapeutic approach would be more beneficial than a conviction [16]. The specific form of these approaches is not made clear in the amendment, but would likely focus on drug-treatment and education programs.

Given the current Government in Aotearoa New Zealand has made it clear that cannabis laws will remain unchanged for the foreseeable future, the current study investigates whether the 2019 amendment has ameliorated the bias in cannabis convictions in Aotearoa New Zealand. Given both the history of Aotearoa New Zealand, and research on discretion and bias in policing [20, 26], we had a single hypothesis: the 2019 amendment would have no impact on the disparity in cannabis convictions between Māori and non-Māori.

## Materials and method

The IDI consists of administrative data including Police, Ministry of Justice (court), and Corrections records. All datasets are linked both deterministically (where possible) and probabilistically (where sufficient information is lacking) on an individual level, allowing us to integrate a multitude of data sources for a single individual.

### Population

We constructed a population in the IDI, defined as anyone who has an administrative record updated within the last 12 months, and who spent more than 12 of the last 16 months in Aotearoa New Zealand. We used a 12-month reference window for the 2018 calendar year (year of the legislative amendment).

### Measures

In the IDI, a population table was constructed that combines demographic information, age, sex, and ethnicity. These data were drawn from several sources, the sources are ranked based on quality/reliability of the source as determined by Stats New Zealand, and we used the highest quality source available. With respect to ethnicity, we formed two ethnic groups—Māori and Pākehā (New Zealanders of European descent). A ‘total response’ approach was used for ethnicity, so that if an individual identified as both ethnic groups it would be explicitly captured in the model.

#### Before and after the amendment

The dummy coded amendment variable was a binary variable where a value of value of 0 represents the 12 months prior to the 13th of August 2019, the date the Misuse of Drugs Act Amendment became active, and 1 is the 12 months after the amendment.

#### Cannabis charge

We took a conservative estimate of a cannabis charge, only including individuals who were charged for possession of cannabis, or cannabis related paraphernalia, for personal use. We took this approach as the amendment does not rule against cannabis-related charges entirely, but instead encourages methods of proceeding outside of the court system. As an example, police could choose to proceed with informal/formal warnings, referrals to health programs, or to drop the charges entirely. In short, the cannabis charge was for an individual who was referred to the courts by police, and was being charged for a personal use of cannabis.

#### Previous charges

As previous criminal charges could impact on whether a cannabis charge is referred to the courts, we added a binary variable for previous charges for any crime, regardless of whether it proceeded to court or was handled out of court.

### Analytical Approach

We used a logistic regression model with cannabis charge as an outcome, with age and previous charges as covariates. We included amendment change and the two ethnicity variables as main effects and, pertinent to our hypothesis, we included interaction terms between each ethnic group and the amendment change variable. The interaction terms allowed us to test whether each ethnic group experienced the same, or significantly different odds in being charged after the amendment was enacted relative to before it was enacted. As we included a near complete population both before and after the amendment, we included random intercepts by individual to account for a repeated measures design. In order to handle the particularly large amount of data, the model was implemented using the mgcv package and parallelized across 20 cores using the parallel package in R [21, 33, 34].

## Results

After constructing the population in the IDI and filtering down to those between 18 and 65 who were Māori or Pākehā, we had a population of 4,600,000 people, of whom 546,879 were Māori and 50% were women (Table 1). The proportion of people who had any cannabis charges that proceeded to the courts was less than 1% across all ethnicities, but due to the large population, counts were adequate for logistic regression as there were over 2,000 charges.

**Table 1:** Descriptive statistics of IDI population

	Pākehā	Māori
*N*	2,198,292	546,879
Age (*M*; SD)	40.82; 14.27	37.07; 14.06
Sex (% female)	50.37%	50.10%
Cannabis charge (pre-amendment–post-amendment)	2151–1692	1473–1299
Previous charge (pre-amendment–post-amendment	1470–1218	1140–1032

The logistic regression models predicting cannabis charges for men and women are shown in Table 2. With respect to the overall number of convictions, there was no effect of the amendment or differing effects of the amendment in the 12 months after the amendment was inacted when compared to the 12 months prior. With respect to our hypothesis, Māori ethnicity was a significant predictor of the odds of receiving a cannabis conviction for Māori males, with a marginal effect for Māori females (Table 2). Further, consistent with our hypothesis, there was no reduction in the number of cannabis charges after the amendment for people of Māori ethnicity (Table 2). In both the male and female models, a previous conviction had a marked impact, likely due to the fact a previous conviction largely eliminates the possibility of a non-conviction pathway.

**Table 2:** Coefficients and statistical summaries from GAMs by gender

	Odds	Std. error	*z* value	*p* value
*Male*				
Intercept	0.00	1.23	− 45.67	**0.000**
Pākehā	1.28	1.20	1.39	0.165
Māori	1.56	1.17	2.89	**0.004**
Previous charge	88,223.05	1.09	128.45	**0.000**
Amendment	0.89	1.31	− 0.44	0.662
Amendment * Pākehā	0.81	1.29	− 0.83	0.408
Amendment * Māori	0.92	1.25	− 0.39	0.698
*Female*				
Intercept	0.00	1.38	− 31.24	**0.000**
Pākehā	1.25	1.35	0.73	0.463
Māori	1.57	1.28	1.84	**0.066**
Previous charge	141,837.84	1.17	76.72	**0.000**
Amendment	1.07	1.58	0.15	0.882
Amendment * Pākehā	0.57	1.53	− 1.31	0.190
Amendment * Māori	0.69	1.43	− 1.04	0.300

Bolded *p* values are considered statistically significant

All analyses adjusted for age

## Discussion

Using data from 4,600,000 people, of whom 546,879 were Māori, we demonstrate that Māori are markedly more likely to be convicted for cannabis possession than Pākehā. This finding is consistent with work on Indigenous people in Canada [2, 19, 23] and Australia [14], demonstrating that cannabis laws have a disproportionate impact on Indigenous peoples in these settler societies. Further, consistent with our hypothesis, the 2019 amendment to the Misuse of Drugs Act had no impact on this racial bias [16]. That is, Māori are still disproportionately impacted by the illegal status of cannabis in Aotearoa New Zealand. Further, criminal convictions are disproportionately given to Maori, whereby the punishment often outweighs the severity of the original offence, and the impact of conviction remains throughout the individual and their family’s life.

Our findings have important implications for the current Government in Aoteroa New Zealand. Following the narrow defeat of the 2020 referendum on the legalisation of cannabis, acting Justice Minister Andrew Little ruled out the possibility of decriminalisation, stating that the Government has “…no other plans for drug law reform” and adding that “The New Zealand voting public have made their decision. We have to respect that decision” [13, 30]. Further, when more than 25 health and social service organisations, including the Māori Law Society and a number of Māori health organisations (e.g., Te Hauora O Turanganui a Kiwa and Te Rau Ora), signed an open letter to the Government, calling for the repeal and replacement of the Misuse of Drugs Act [10], Minister Little stated that “their gesture today is 12 months too late" [13].

Despite Minister Little’s assertion that the open-letter was simply a gesture, and too late, the referendum is only a reflection of the public’s views on the Cannabis Legalisation and Control Bill, which had a specific focus on creating a legal market for cannabis [3]. Thus, it is incorrect to suggest it reflects the public’s views on cannabis decriminalisation. On that front, the only information we have are public polls. For example, a recent poll of people who voted no in the referendum (i.e., against legalisation), revealed that just under half were supportive of decriminalisation [24]. Thus, when considered in combination with the extremely close referendum result (48.4% for, 50.7% against), it seems a more accurate to argue that a sizable portion of the public supports decriminalisation.

## Conclusion

Following the referendum, when asked about further drug law reform, Prime Minister Jacinda Ardern said that her government would "drill into that data" to investigate whether the 2019 amendment was having a positive impact. The current study has done that drilling, and revealed that the 2019 amendment continues to disproportionately impact Māori. Our findings, when combined with the United Nations Declaration on the Rights of Indigenous Peoples and Te Tiriti o Waitangi, give the government no choice but to move forward on drug law reform.

## Data Availability

The data used in this study are held with the Integrated Data Infrastructure and are managed by Statistics New Zealand. These data are publicly available, although access is restricted (See https://www.stats.govt.nz). These results are not official statistics. They have been created for research purposes from the Integrated Data Infrastructure (IDI) which is carefully managed by Stats NZ. For more information about the IDI please visit https://www.stats.govt.nz/integrated-data/.
